# Crystal structure and Hirshfeld surface analysis of 4-allyl-2-meth­oxy-6-nitro­phenol

**DOI:** 10.1107/S2056989020002601

**Published:** 2020-02-28

**Authors:** Yassine El Ghallab, Sanae Derfoufi, El Mostafa Ketatni, Mohamed Saadi, Lahcen El Ammari

**Affiliations:** a Laboratory of Drugs Sciences, Biomedical Research and Biotechnology, Faculty of Medicine and Pharmacy, Hassan II University, BP 9154, Casablanca 20250, Morocco; b Laboratory of Organic and Analytical Chemistry, University Sultan Moulay, Slimane, Faculty of Science and Technology, PO Box 523, Beni-Mellal, Morocco; c Laboratoire de Chimie Appliquée des Matériaux, Centre des Sciences des Matériaux, Faculty of Sciences, Mohammed V University in Rabat, Avenue Ibn Batouta, BP 1014, Rabat, Morocco

**Keywords:** crystal structure, nitro­eugenol, hydrogen bonds, Hirshfeld surface analysis, IR, NMR

## Abstract

The asymmetric unit of the title compound, C_10_H_11_NO_4_, which was synthesized *via* nitration reaction of eugenol (4-allyl-2-meth­oxy­phenol) with a mixture of nitric acid and sulfuric acid, consists of three independent mol­ecules of similar geometry. Each mol­ecule displays an intra­molecular hydrogen bond involving the hydroxide and the nitro group forming an *S*(6) motif. The crystal cohesion is ensured by inter­molecular C—H⋯O hydrogen bonds in addition to π–π stacking inter­actions between the aromatic rings [centroid–centroid distances = 3.6583 (17)–4.0624 (16) Å]. The Hirshfeld surface analysis and the two-dimensional fingerprint plots show that H⋯H (39.6%), O⋯H/H⋯O (37.7%), C⋯H/H⋯C (12.5%) and C⋯C (4%) are the most important contributors towards the crystal packing.

## Abstract 2

The crystal structure of 4-allyl-2-meth­oxy-6-nitro­phenol, which crystallizes in the centrosymmetric space group *P* 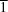 with three independent mol­ecules in the asymmetric unit, is reported along with the Hirshfeld surface analysis.

## Chemical context   

Eugenol, the main constituent of clove essential oil, has many inter­esting biological properties and participates in the synthesis of bioactive compounds (Kaufman, 2015 ▸). The nitro­eugenol isomers were tested for their anti­fungal activity, growth inhibitory activity on human tumor cell lines (Carrasco *et al.*, 2012 ▸, 2008 ▸), and anti­oxidant activity (Hidalgo *et al.*, 2009 ▸). We report here the synthesis, structure, spectrometric and spectroscopic characterization of the title compound along with an analysis of the calculated Hirshfeld surface and the two-dimensional fingerprint plots.
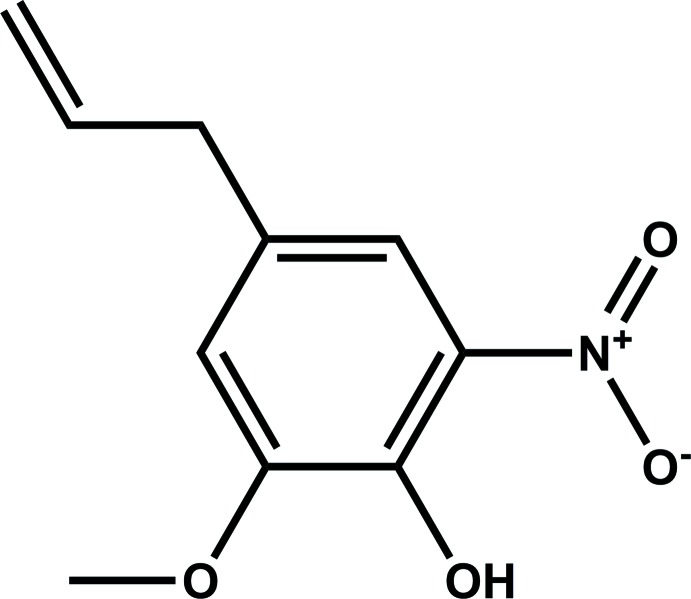


## Structural commentary   

**Figure 1:**
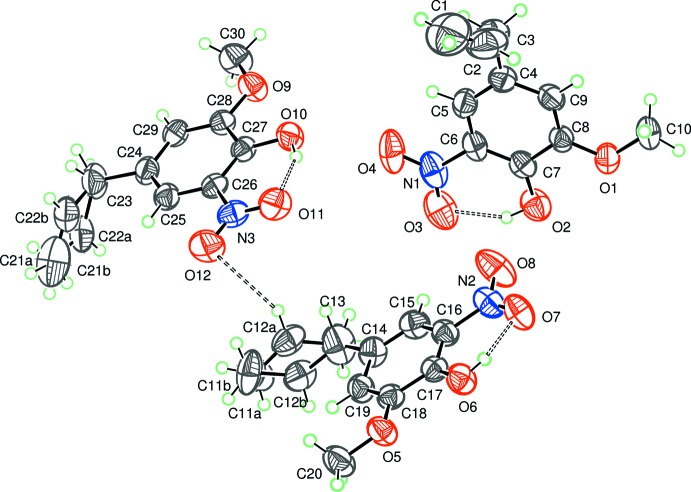
The asymmetric unit of the title compound with the displacement ellipsoids drawn at the 50% probability level. H atoms are represented as small circles. Intra- and inter­molecular hydrogen bonds are shown as dashed lines.

The asymmetric unit of the title compound (Fig. 1 ▸) contains three independent mol­ecules of similar geometry hereafter referred as Mol-N1 (N1/O1–O4/C1–C10), Mol-N2 (N2/O5–O8/C11–C20) and Mol-N3 (N3/O9–O12/C21–C30). The planes through the nitro groups are almost coplanar with those of the attached benzene rings, forming dihedral angles ranging from 2.1 (3)° in Mol-N3 to 6.38 (13)° in Mol-N2. The mean planes though the allyl group C1/C2/C3 (mol­ecule Mol-N1) and the disordered allyl groups C11*A*/C11*B*/C12*A*/C12*B*/C13 (mol­ecule Mol-N2) and C21*A*/C21*B*/C22*A*/C22*B*/C23 (mol­ecule Mol-N3) are oriented with dihedral angles of 67.5 (3), 80.8 (3) and 86.1 (4)°, respectively, to the attached benzene rings. The benzene rings of mol­ecules Mol-N2 and Mol-N3 are approximately parallel to each other [dihedral angle 10.60 (7)°], and roughly perpendicular to that of Mol-N1 [dihedral angles of 83.65 (7) and 79.22 (6)°, respectively]. A strong intra­molecular O—H⋯O hydrogen bond involving a nitro O atom and the H atom of the hydroxide group forming an *S*(6) motif is observed in each mol­ecule (Table 1 ▸).

## Supra­molecular features   

**Figure 2:**
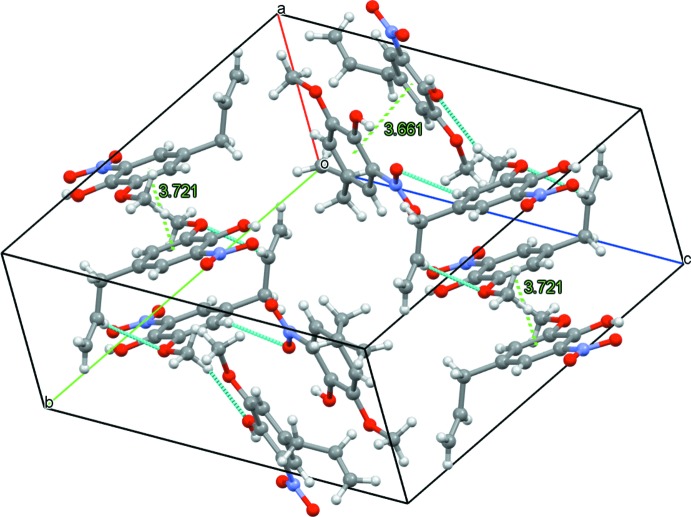
Partial crystal packing of the title compound showing mol­ecules connected by hydrogen bonds (dashed cyan lines) and π–π inter­actions (dashed green lines).

In the crystal, the mol­ecules are connected by inter­molecular C12*A*—H12*A*⋯O12, C12*B*—H12*B*⋯O3 and C9—H9⋯O7 hydrogen bonds (Table 1 ▸; Figs. 2 ▸ and 3 ▸). In addition, centrosymmetrically related pairs of Mol-N1 mol­ecules are connected by π–π inter­actions to form dimeric units [centroid–centroid distance = 3.7213 (15) Å] (Fig. 2 ▸), whereas the Mol-N2 and Mol-N3 mol­ecules are stacked through π–π inter­actions to form chains running parallel to the *b* axis [*Cg*2⋯*Cg*2^i^ = 3.6583 (17) Å; *Cg*2⋯*Cg*3^ii^ = 3.6613 (18) Å; *Cg*3⋯*Cg*3^iii^ = 4.0624 (16) Å; symmetry codes: (i) 2 − *x*, 1 − *y*, 1 − *z*; (ii) 1 + *x*, *y*, *z*; (iii) −*x*, −*y*, 1 − *z*].

## Hirshfeld surface analysis   

**Figure 4:**
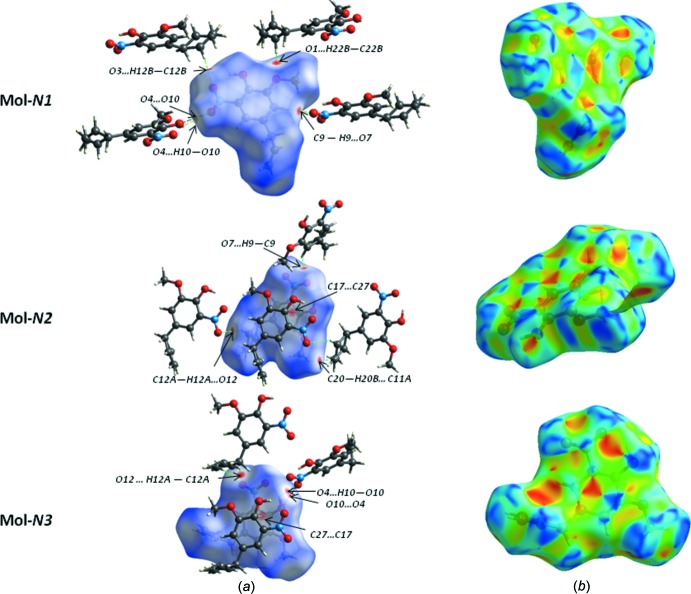
Hirshfeld surface of the title compound (symmetry-independent mol­ecules Mol-N1, Mol-N2 and Mol-N3), with (*a*) *d*_norm_ with the inter­action of neighbouring mol­ecules and (*b*) shape-index.

**Figure 5:**
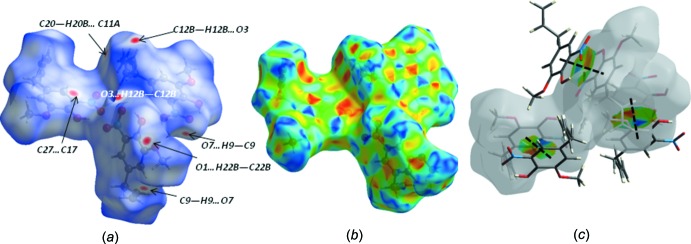
Views of the Hirshfeld surface for a reference mol­ecule of the title compound mapped over (*a*) *d*_norm_, (*b*) shape-index and (*c*) the shape-index property highlighting the π–π inter­actions as black dashed lines.

**Figure 6:**
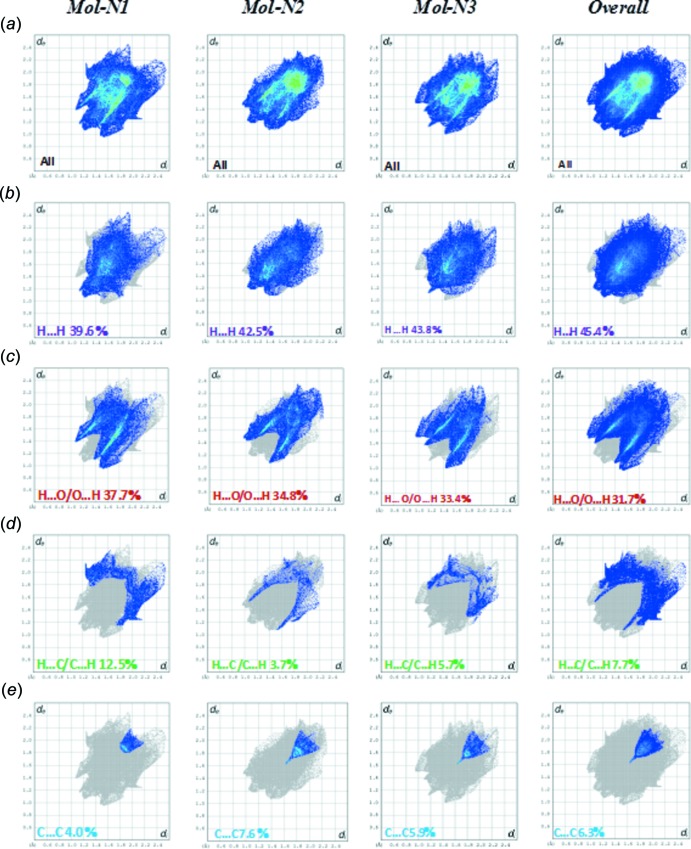
Fingerprint plots representative of specific inter­atomic contacts in the title compound (symmetry-independent mol­ecules Mol-N1, Mol-N2, Mol-N3 and overall), delineated into H⋯H, O⋯H/H⋯O, C⋯H/H⋯C and C⋯C inter­actions.

**Figure 7:**
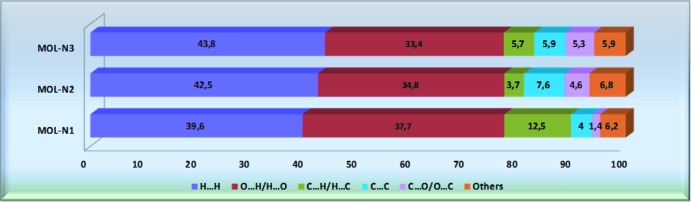
Percentage contribution of various inter­molecular inter­actions in the title compound obtained from decomposed fingerprint plots.

In order to explore the nature of the inter­molecular contacts and their role in the crystal packing, Hirshfeld surfaces (Spackman & Jayatilaka, 2009 ▸) and the associated two-dimensional fingerprint plots (McKinnon *et al.*, 2007 ▸) were calculated using *Crystal Explorer 17.5* (Turner *et al.*, 2017 ▸). The three-dimensional mol­ecular Hirshfeld surfaces of the three mol­ecules Mol-N1, Mol-N2 and Mol-N3 and the overall surface were generated using a high standard surface resolution colour-mapped over the normalized contact distance. The red, white and blue regions visible on the *d*_norm_ surfaces indicate contacts with distances shorter, longer and equal to the van der Waals radii (Fig. 4 ▸ *a* and 5 ▸ *a*). The shape-index of the Hirshfeld surface is a tool to visualize the π–π stacking inter­actions (Fig. 4 ▸ *b* and 5*b*). The red spots in Fig. 4 ▸ *a* correspond to the strong C—H⋯O hydrogen-bond inter­actions in the crystal structure; in Mol-N1 two of them involve the O atoms of the meth­oxy (O1) and nitro (O3) groups as acceptors with allyl H atoms (C22*B*– H22*B*⋯O1 and C12*B*—H12*B*⋯O3), while the other is due to the inter­atomic inter­action between the aromatic H9 donor atom and the nitro O7 oxygen atom (C9—H9⋯O7). The longer O—H⋯O hydrogen bonds and O⋯O inter­actions are characterized by smaller red spots close to each other on the surface, where the faint red spot indicating the O—H⋯O inter­actions is associated with the longest O⋯O contact of 2.96 (3) Å in Mol-N1 and Mol-N3. In Mol-N2, the red spots correspond to C—H⋯O (C9—H9⋯O7 and C12*A*—H12*A*⋯O12) and C—H⋯C (C20—H20*B*⋯C11*A*) hydrogen-bond inter­actions. The corresponding fingerprint plots for each of the independent mol­ecules and for the entire asymmetric unit, showing characteristic pseudo-symmetric wings in the *d*_e_ and *d*_i_ diagonal axes, and those delineated into H⋯H, O⋯H/H⋯O, C⋯H/H⋯C and C⋯C contacts are illustrated in Fig. 6 ▸. The result of the qu­anti­tative analysis of all types of inter­molecular contacts present in the title compound is summarized in Fig. 7 ▸. The most important inter­action is H⋯H, contributing 45.4% to the overall crystal packing (Fig. 6 ▸ *b*), which is reflected in the widely scattered points of high density due to the large hydrogen-atom content of the mol­ecule. The contribution from the O⋯H/H⋯O contacts (31.7%), corresponding to C—H⋯O and O—H⋯O inter­actions, is represented by a pair of sharp spikes characteristic of a strong hydrogen-bond inter­action with *d*_e_ + *d*_i_ ≃ 2.5Å (Fig. 6 ▸ *c*). In the absence of weak C—H⋯π inter­actions in the crystal, the pair of characteristic wings in the fingerprint plot delineated into H⋯C/C⋯H contacts (7.7% contribution) have a symmetrical distribution of points (Fig. 6 ▸ *d*), with the tips at *d*_e_ + *d*_i_ ≃ 2.65 Å. The distribution of points in the *d*_e_ = *d*_i_ ≃ 1.6 Å range in the fingerprint plot delineated into C⋯C contacts (Fig. 6 ▸ *e*) indicates the existence of weak π–π stacking inter­actions between the phenyl rings, which are indicated by adjacent red and blue triangles in the shape-index map (Fig. 4 ▸ *b* and Fig. 5 ▸ *c*). The small contribution of the other weak inter­molecular O⋯O, N⋯H/H⋯N, C⋯O/O⋯C, C⋯N/N⋯C and N⋯O/O⋯N contacts has a negligible effect on the packing.

## Database survey   

A search of the Cambridge Structural Database (CSD, Version 5.40, May 2019; Groom *et al.*, 2016 ▸) of eugenol derivatives revealed two compounds with very similar structures but with a different position of the nitro group or with the hydroxide group substituted by an acetate group, *viz.* 4-allyl-2-meth­oxy-5-nitro­phenyl acetate (refcode: TEJREG; Carrasco-Altamirano *et al.*, 2006 ▸) and 4-hy­droxy-3-meth­oxy-5-nitro­aceto­phenone (5-nitro­apocynin) (MUCDOE; Babu *et al.*, 2009 ▸). A third related compound, 4-hy­droxy-3-meth­oxy-5-nitro­benzaldehyde, has recently been reported (Vusak *et al.*, 2020 ▸). All of these compounds exhibit intra­molecular hydrogen bonds involving the nitro O atoms with the H atoms of the hydroxide group, and other inter­molecular hydrogen bonds, in addition to π–π inter­actions, which assure the crystal cohesion.

## Synthesis and crystallization   

In a 250 mL flat-bottom flask containing a stirred solution of eugenol (2.12 g, 12.9 mmol) and di­chloro­methane (60 mL), a mixture of concentrated sulfuric acid (0.78 mL) and concentrated nitric acid (0.80 mL) was added dropwise for 30 min at 273 K. The complete disappearance of the starting product was confirmed by means of thin layer chromatography using *n*-hexa­ne/AcOEt (9:1 *v*/*v*) as eluent. The reaction mixture was diluted with di­chloro­methane, washed with brine (3 × 10 mL), dried over anhydrous Na_2_SO_4_ and concentrated under vacuum. The crude product was subjected to chromatography on a silica-gel column with *n*-hexa­ne/AcOEt (9:1 *v*/*v*) as eluent to afford the title compound as a reddish-orange liquid. Reddish-orange crystals formed spontaneously with a yield of 56%. Good quality crystals suitable for single crystal X-ray diffraction analysis were obtained by slow evaporation of an *n*-hexa­ne:AcOEt solution, m.p. = 317–319 K.

**Figure 8:**
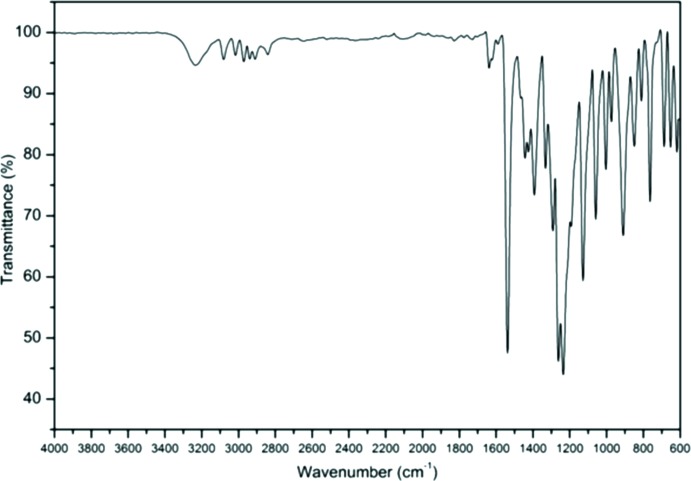
The FT–IR spectrum of the title compound.

IR (cm^−1^): 3235, 3080, 3016, 2971, 2910, 1638, 1537, 1392, 1331, 1262, 1128, 1059, 909, 763. The FT–IR spectrum (Fig. 8 ▸) illustrates several bands characteristic of 4-allyl-2-meth­oxy-6-nitro­phenol. The absorption band at 3235 cm^−1^ was assigned to the O—H stretching vibration. The bands located at 3080 and 3016 cm^−1^ correspond to the C=CH bond of the aromatic ring and CH=CH_2_ bond of the allyl group, respectively. The remarkably strong band at 1537 cm^−1^ was attributed to the stretching vibration of the nitro group. Other C=C stretching vibrations are at 2971, 2910 and 1638 cm^−1^. The FT–IR spectrum peaks are in agreement with the reported data for similar compounds (Carrasco *et al.*, 2008 ▸; Egorov *et al.*, 2014 ▸; Heredia *et al.*, 2016 ▸).

^1^H NMR (CDCl_3_, 300 MHz) δ 10.7 (*s*, 1H, OH conjugated), 7.53 (*s*, 1H, Ar-H), 6.99 (*s*, 1H, Ar-H), 6.01–5.88 (*m*, 1H), 5.19–5.13 (*m*, 2H), 3.96 (*s*, 3H), 3.39–3.37 (*d*, 2H). ^13^C NMR (CDCl_3_, 75.5 MHz) δ 149.87, 144.90, 135.95, 133.66, 131.24, 118.63, 117.16, 115.11, 114.29, 56.72, 39.41. FTMS–ESI, *m*/*z*: 208.04616 (100%) [C_10_H_11_NO_4_].

## Refinement   

Crystal data, data collection and structure refinement details are summarized in Table 2 ▸. The C-bound H atoms were located in a difference-Fourier map and refined as riding with C—H = 0.93–0.97 Å, and *U*_iso_(H) = 1.2 *U*_eq_(C) or 1.5*U*_eq_(C) for methyl H atoms. A rotating model was used for the methyl groups. The hydroxyl H atoms were located in a difference-Fourier map and refined freely. The two allyl groups of Mol-N2 and Mol-N3 are disordered over two sets of sites with refined occupancy ratios of 0.648 (8):0.352 (8) and 0.668 (9):0.332 (9) respectively. One outlier (100) was omitted in the cycles of refinement.

## Supplementary Material

Crystal structure: contains datablock(s) I. DOI: 10.1107/S2056989020002601/rz5270sup1.cif

Structure factors: contains datablock(s) I. DOI: 10.1107/S2056989020002601/rz5270Isup2.hkl

Supporting information file. DOI: 10.1107/S2056989020002601/rz5270Isup3.cml

CCDC reference: 1986157

Additional supporting information:  crystallographic information; 3D view; checkCIF report

## Figures and Tables

**Figure 3:**
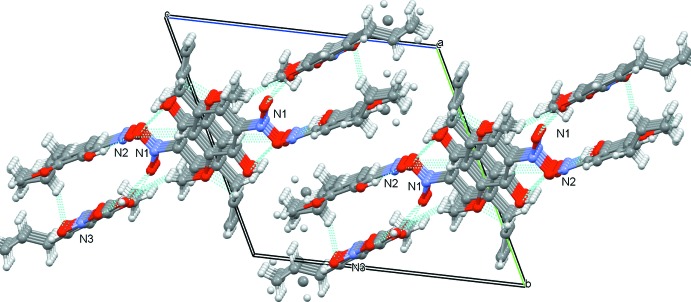
Crystal packing of the title compound viewed along the *a* axis showing mol­ecules linked by hydrogen bonds (dashed cyan lines).

**Table 1:** Hydrogen-bond geometry (Å, °)

*D*—H⋯*A*	*D*—H	H⋯*A*	*D*⋯*A*	*D*—H⋯*A*
O10—H10⋯O11	0.86 (3)	1.81 (3)	2.594 (2)	149 (2)
O6—H6⋯O7	0.83 (2)	1.83 (2)	2.584 (2)	152 (2)
O2—H2*O*⋯O3	0.91 (3)	1.78 (3)	2.587 (2)	146 (2)
C12*A*—H12*A*⋯O12	0.93	2.58	3.382 (4)	145
C12*B*—H12*B*⋯O3^i^	0.93	2.56	3.325 (8)	140
C9—H9⋯O7^ii^	0.93	2.59	3.394 (2)	145

Symmetry codes: (i) 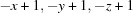; (ii) 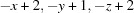.

**Table 2:** Experimental details

Crystal data	
Chemical formula	C_10_H_11_NO_4_
*M*_r_	209.20
Crystal system, space group	Triclinic, *P* 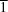
Temperature (K)	296
*a*, *b*, *c* (Å)	8.706 (3), 13.753 (5), 14.683 (5)
α, β, γ (°)	116.142 (11), 93.871 (12), 96.985 (12)
*V* (Å^3^)	1552.0 (9)
*Z*	6
Radiation type	Mo *K*α
μ (mm^−1^)	0.11
Crystal size (mm)	0.31 × 0.28 × 0.26

Data collection	
Diffractometer	Bruker D8 VENTURE Super DUO
Absorption correction	Multi-scan (*SADABS*; Krause *et al.*, 2015 ▸)
*T*_min_, *T*_max_	0.707, 0.746
No. of measured, independent and observed [*I* > 2σ(*I*)] reflections	54955, 6334, 4687
*R*_int_	0.037
(sin θ/λ)_max_ (Å^−1^)	0.625

Refinement	
*R*[*F* ^2^ > 2σ(*F* ^2^)], *wR*(*F* ^2^), *S*	0.047, 0.134, 1.02
No. of reflections	6334
No. of parameters	460
H-atom treatment	H atoms treated by a mixture of independent and constrained refinement
Δρ_max_, Δρ_min_ (e Å^−3^)	0.41, −0.20

Computer programs: *APEX3* and *SAINT* (Bruker, 2016 ▸), *SHELXT2014/5* (Sheldrick, 2015*a* ▸), *SHELXL2016/6* (Sheldrick, 2015*b* ▸), *ORTEP-3 for Windows* (Farrugia, 2012 ▸); *Mercury* (Macrae *et al.*, 2020 ▸) and *publCIF* (Westrip, 2010 ▸).

## supplementary crystallographic information

### Crystal data

**Table:**

C_10_H_11_NO_4_	*Z* = 6
*M**_r_* = 209.20	*F*(000) = 660
Triclinic, *P*1	*D*_x_ = 1.343 Mg m^−^^3^
*a* = 8.706 (3) Å	Mo *K*α radiation, λ = 0.71073 Å
*b* = 13.753 (5) Å	Cell parameters from 6334 reflections
*c* = 14.683 (5) Å	θ = 2.7–26.4°
α = 116.142 (11)°	µ = 0.11 mm^−^^1^
β = 93.871 (12)°	*T* = 296 K
γ = 96.985 (12)°	Block, orange
*V* = 1552.0 (9) Å^3^	0.31 × 0.28 × 0.26 mm

### Data collection

**Table:**

Bruker D8 VENTURE Super DUO diffractometer	6334 independent reflections
Radiation source: INCOATEC IµS micro-focus source	4687 reflections with *I* > 2σ(*I*)
HELIOS mirror optics monochromator	*R*_int_ = 0.037
Detector resolution: 10.4167 pixels mm^-1^	θ_max_ = 26.4°, θ_min_ = 2.7°
φ and ω scans	*h* = −10→10
Absorption correction: multi-scan (SADABS; Krause *et al.*, 2015)	*k* = −17→17
*T*_min_ = 0.707, *T*_max_ = 0.746	*l* = −18→18
54955 measured reflections	

### Refinement

**Table:**

Refinement on *F*^2^	Hydrogen site location: mixed
Least-squares matrix: full	H atoms treated by a mixture of independent and constrained refinement
*R*[*F*^2^ > 2σ(*F*^2^)] = 0.047	*w* = 1/[σ^2^(*F*_o_^2^) + (0.0592*P*)^2^ + 0.4096*P*] where *P* = (*F*_o_^2^ + 2*F*_c_^2^)/3
*wR*(*F*^2^) = 0.134	(Δ/σ)_max_ < 0.001
*S* = 1.02	Δρ_max_ = 0.41 e Å^−^^3^
6334 reflections	Δρ_min_ = −0.20 e Å^−^^3^
460 parameters	Extinction correction: SHELXL-2018/3 (Sheldrick, 2015b), Fc^*^=kFc[1+0.001xFc^2^λ^3^/sin(2θ)]^-1/4^
0 restraints	Extinction coefficient: 0.021 (2)

### Special details

**Table:**

Geometry. All esds (except the esd in the dihedral angle between two l.s. planes) are estimated using the full covariance matrix. The cell esds are taken into account individually in the estimation of esds in distances, angles and torsion angles; correlations between esds in cell parameters are only used when they are defined by crystal symmetry. An approximate (isotropic) treatment of cell esds is used for estimating esds involving l.s. planes.

### Fractional atomic coordinates and isotropic or equivalent isotropic displacement parameters (Å^2^)

**Table:**

	*x*	*y*	*z*	*U*_iso_*/*U*_eq_	Occ. (<1)
C1	0.7703 (5)	0.1282 (3)	0.9575 (3)	0.1442 (14)	
H1A	0.669741	0.087821	0.935152	0.173*	
H1B	0.856141	0.092334	0.943266	0.173*	
C2	0.7907 (4)	0.2334 (3)	1.0082 (2)	0.1086 (9)	
H2	0.893725	0.269409	1.028622	0.130*	
C3	0.6708 (3)	0.30301 (19)	1.03745 (18)	0.0862 (7)	
H3A	0.681015	0.341956	1.111572	0.103*	
H3B	0.568772	0.256699	1.013201	0.103*	
C4	0.6796 (2)	0.38578 (15)	0.99543 (14)	0.0619 (5)	
C5	0.5910 (2)	0.36410 (16)	0.90633 (14)	0.0628 (5)	
H5	0.523012	0.297687	0.870481	0.075*	
C6	0.60267 (19)	0.44162 (15)	0.86912 (13)	0.0564 (4)	
C7	0.7021 (2)	0.54237 (14)	0.92020 (13)	0.0533 (4)	
C8	0.79192 (19)	0.56398 (14)	1.01284 (12)	0.0516 (4)	
C9	0.7813 (2)	0.48670 (15)	1.04822 (13)	0.0576 (4)	
H9	0.842850	0.501427	1.108578	0.069*	
C10	0.9739 (3)	0.69329 (18)	1.15663 (15)	0.0763 (6)	
H10A	0.904755	0.692601	1.204647	0.115*	
H10B	1.034076	0.765533	1.181861	0.115*	
H10C	1.042918	0.641401	1.148116	0.115*	
N1	0.50602 (19)	0.41416 (17)	0.77400 (13)	0.0741 (5)	
O1	0.88445 (16)	0.66397 (10)	1.06010 (10)	0.0687 (4)	
O2	0.71821 (19)	0.62197 (12)	0.89023 (11)	0.0745 (4)	
O3	0.5206 (2)	0.47908 (17)	0.73564 (13)	0.1008 (6)	
O4	0.41380 (18)	0.32741 (15)	0.73302 (13)	0.0988 (6)	
C11A	0.4789 (14)	0.2126 (11)	0.2212 (9)	0.107 (4)	0.648 (8)
H11A	0.504547	0.263438	0.196542	0.128*	0.648 (8)
H11B	0.429013	0.141711	0.176573	0.128*	0.648 (8)
C12A	0.5127 (4)	0.2406 (3)	0.3175 (3)	0.0775 (16)	0.648 (8)
H12A	0.484720	0.186924	0.338427	0.093*	0.648 (8)
C11B	0.472 (2)	0.1982 (15)	0.2141 (18)	0.094 (7)	0.352 (8)
H11C	0.441218	0.141392	0.230507	0.113*	0.352 (8)
H11D	0.453749	0.186675	0.146615	0.113*	0.352 (8)
C12B	0.5447 (7)	0.3007 (8)	0.2904 (7)	0.075 (3)	0.352 (8)
H12B	0.570414	0.351468	0.265767	0.090*	0.352 (8)
C13	0.5863 (3)	0.3424 (3)	0.3925 (2)	0.1000 (8)	
H13A	0.527879	0.363796	0.450327	0.120*	0.648 (8)
H13B	0.581070	0.395340	0.365880	0.120*	0.648 (8)
H13C	0.522486	0.297742	0.416294	0.120*	0.352 (8)
H13D	0.559627	0.415525	0.424614	0.120*	0.352 (8)
C14	0.7560 (2)	0.35083 (16)	0.43148 (15)	0.0619 (5)	
C15	0.8104 (2)	0.39916 (15)	0.53351 (15)	0.0610 (5)	
H15	0.742814	0.427603	0.581481	0.073*	
C16	0.9676 (2)	0.40594 (13)	0.56597 (13)	0.0532 (4)	
C17	1.07440 (19)	0.36536 (13)	0.49776 (13)	0.0493 (4)	
C18	1.01636 (19)	0.31557 (14)	0.39249 (12)	0.0507 (4)	
C19	0.8614 (2)	0.30891 (15)	0.36115 (14)	0.0568 (4)	
H19	0.825339	0.275728	0.291428	0.068*	
C20	1.0751 (3)	0.2270 (2)	0.22242 (15)	0.0808 (6)	
H20A	0.995975	0.163994	0.203937	0.121*	
H20B	1.162250	0.204457	0.186106	0.121*	
H20C	1.033249	0.278869	0.204844	0.121*	
N2	1.0193 (2)	0.45748 (14)	0.67507 (12)	0.0697 (4)	
O5	1.12547 (14)	0.27684 (12)	0.32966 (9)	0.0664 (4)	
O6	1.22701 (14)	0.36805 (12)	0.52235 (11)	0.0641 (4)	
O7	1.1606 (2)	0.47349 (13)	0.70603 (11)	0.0850 (4)	
O8	0.9228 (2)	0.48412 (17)	0.73412 (12)	0.1068 (6)	
C21A	−0.2510 (12)	−0.0879 (7)	0.0621 (5)	0.121 (4)	0.668 (9)
H21A	−0.246926	−0.160899	0.045748	0.145*	0.668 (9)
H21B	−0.232777	−0.062093	0.014302	0.145*	0.668 (9)
C22A	−0.2799 (4)	−0.0266 (4)	0.1454 (3)	0.0764 (14)	0.668 (9)
H22A	−0.281443	0.044748	0.154888	0.092*	0.668 (9)
C21B	−0.233 (2)	−0.0934 (12)	0.0612 (16)	0.119 (8)	0.332 (9)
H21C	−0.233452	−0.028161	0.056564	0.143*	0.332 (9)
H21D	−0.206887	−0.154152	0.007319	0.143*	0.332 (9)
C22B	−0.2705 (8)	−0.0999 (9)	0.1445 (7)	0.078 (3)	0.332 (9)
H22B	−0.264145	−0.170393	0.135978	0.094*	0.332 (9)
C23	−0.3132 (3)	−0.0433 (2)	0.23518 (18)	0.0893 (7)	
H23A	−0.323904	−0.121250	0.215662	0.107*	0.668 (9)
H23B	−0.412887	−0.021186	0.253318	0.107*	0.668 (9)
H23C	−0.385321	−0.093840	0.248213	0.107*	0.332 (9)
H23D	−0.371941	0.009953	0.229832	0.107*	0.332 (9)
C24	−0.1915 (2)	0.01826 (14)	0.32956 (14)	0.0575 (4)	
C25	−0.0350 (2)	0.01984 (14)	0.32198 (13)	0.0547 (4)	
H25	−0.001961	−0.015992	0.258299	0.066*	
C26	0.07431 (18)	0.07529 (13)	0.40997 (13)	0.0483 (4)	
C27	0.03114 (18)	0.13021 (13)	0.50680 (12)	0.0468 (4)	
C28	−0.13073 (18)	0.12718 (13)	0.51315 (13)	0.0494 (4)	
C29	−0.23785 (19)	0.07271 (14)	0.42663 (13)	0.0538 (4)	
H29	−0.343672	0.071824	0.432412	0.065*	
C30	−0.3235 (2)	0.1869 (2)	0.62435 (17)	0.0773 (6)	
H30A	−0.364262	0.226379	0.590885	0.116*	
H30B	−0.331320	0.224162	0.696188	0.116*	
H30C	−0.382519	0.113848	0.595437	0.116*	
N3	0.23790 (17)	0.07279 (13)	0.39773 (13)	0.0618 (4)	
O9	−0.16410 (14)	0.18116 (12)	0.61035 (9)	0.0671 (4)	
O10	0.12736 (15)	0.18700 (11)	0.59525 (10)	0.0635 (3)	
O11	0.33723 (15)	0.11885 (14)	0.47501 (12)	0.0795 (4)	
O12	0.27365 (17)	0.02443 (14)	0.31208 (13)	0.0892 (5)	
H2O	0.658 (3)	0.593 (2)	0.829 (2)	0.110 (9)*	
H6	1.236 (3)	0.3989 (19)	0.5856 (19)	0.083 (8)*	
H10	0.218 (3)	0.180 (2)	0.5758 (19)	0.095 (8)*	

### Atomic displacement parameters (Å^2^)

**Table:**

	*U*^11^	*U*^22^	*U*^33^	*U*^12^	*U*^13^	*U*^23^
C1	0.187 (4)	0.103 (2)	0.156 (3)	0.034 (2)	0.036 (3)	0.066 (2)
C2	0.104 (2)	0.135 (3)	0.124 (2)	0.0102 (19)	0.0140 (18)	0.094 (2)
C3	0.1077 (18)	0.0779 (14)	0.0734 (14)	−0.0027 (13)	0.0214 (13)	0.0380 (12)
C4	0.0659 (11)	0.0605 (11)	0.0546 (10)	0.0049 (9)	0.0180 (9)	0.0220 (9)
C5	0.0520 (10)	0.0601 (11)	0.0576 (11)	−0.0001 (8)	0.0113 (8)	0.0118 (9)
C6	0.0435 (9)	0.0666 (11)	0.0460 (9)	0.0157 (8)	0.0047 (7)	0.0124 (8)
C7	0.0523 (9)	0.0584 (10)	0.0486 (9)	0.0199 (8)	0.0120 (7)	0.0203 (8)
C8	0.0486 (9)	0.0534 (9)	0.0450 (9)	0.0077 (7)	0.0091 (7)	0.0151 (7)
C9	0.0595 (10)	0.0661 (11)	0.0434 (9)	0.0078 (8)	0.0075 (7)	0.0220 (8)
C10	0.0808 (14)	0.0703 (13)	0.0558 (11)	−0.0058 (10)	−0.0092 (10)	0.0160 (10)
N1	0.0526 (9)	0.0891 (13)	0.0605 (10)	0.0260 (9)	−0.0019 (8)	0.0139 (10)
O1	0.0777 (9)	0.0581 (7)	0.0579 (7)	−0.0041 (6)	−0.0057 (6)	0.0210 (6)
O2	0.0933 (11)	0.0682 (9)	0.0649 (9)	0.0219 (7)	0.0013 (8)	0.0320 (7)
O3	0.0982 (12)	0.1215 (14)	0.0802 (11)	0.0284 (10)	−0.0179 (9)	0.0452 (11)
O4	0.0648 (9)	0.1008 (12)	0.0829 (11)	0.0057 (9)	−0.0214 (8)	0.0052 (9)
C11A	0.071 (7)	0.169 (11)	0.086 (6)	0.015 (6)	0.001 (5)	0.066 (7)
C12A	0.0454 (17)	0.089 (3)	0.109 (4)	0.0035 (17)	0.0061 (18)	0.058 (3)
C11B	0.047 (8)	0.072 (6)	0.116 (13)	0.002 (5)	−0.011 (7)	0.005 (7)
C12B	0.048 (3)	0.098 (6)	0.095 (6)	0.006 (3)	−0.009 (3)	0.063 (5)
C13	0.0515 (12)	0.129 (2)	0.0964 (19)	0.0272 (13)	0.0084 (12)	0.0283 (16)
C14	0.0487 (10)	0.0655 (11)	0.0684 (12)	0.0144 (8)	0.0089 (8)	0.0262 (9)
C15	0.0617 (11)	0.0596 (10)	0.0645 (11)	0.0196 (8)	0.0200 (9)	0.0267 (9)
C16	0.0638 (11)	0.0497 (9)	0.0492 (9)	0.0134 (8)	0.0077 (8)	0.0243 (8)
C17	0.0489 (9)	0.0491 (9)	0.0547 (9)	0.0089 (7)	0.0046 (7)	0.0281 (8)
C18	0.0457 (9)	0.0558 (9)	0.0537 (10)	0.0080 (7)	0.0093 (7)	0.0274 (8)
C19	0.0479 (9)	0.0638 (11)	0.0542 (10)	0.0076 (8)	0.0029 (7)	0.0237 (8)
C20	0.0663 (13)	0.1128 (18)	0.0546 (11)	0.0144 (12)	0.0145 (9)	0.0295 (12)
N2	0.0902 (13)	0.0670 (10)	0.0547 (9)	0.0267 (9)	0.0126 (9)	0.0265 (8)
O5	0.0479 (7)	0.0950 (10)	0.0525 (7)	0.0144 (6)	0.0107 (5)	0.0287 (7)
O6	0.0518 (7)	0.0819 (9)	0.0592 (8)	0.0140 (6)	0.0006 (6)	0.0327 (7)
O7	0.0913 (11)	0.0988 (11)	0.0571 (8)	0.0192 (9)	−0.0071 (8)	0.0300 (8)
O8	0.1188 (14)	0.1410 (16)	0.0609 (9)	0.0613 (12)	0.0319 (9)	0.0334 (10)
C21A	0.134 (6)	0.142 (7)	0.042 (3)	−0.033 (5)	−0.013 (3)	0.021 (4)
C22A	0.075 (2)	0.061 (3)	0.072 (3)	0.0025 (17)	−0.0183 (16)	0.0174 (19)
C21B	0.112 (11)	0.054 (7)	0.160 (19)	0.039 (8)	−0.008 (9)	0.019 (8)
C22B	0.070 (4)	0.069 (6)	0.071 (5)	−0.001 (4)	−0.005 (3)	0.014 (4)
C23	0.0632 (13)	0.1015 (17)	0.0703 (14)	−0.0118 (12)	−0.0097 (11)	0.0184 (13)
C24	0.0488 (9)	0.0548 (10)	0.0595 (10)	−0.0009 (7)	0.0012 (8)	0.0207 (8)
C25	0.0538 (10)	0.0506 (9)	0.0537 (10)	0.0057 (7)	0.0103 (8)	0.0188 (8)
C26	0.0398 (8)	0.0496 (9)	0.0610 (10)	0.0078 (7)	0.0099 (7)	0.0297 (8)
C27	0.0411 (8)	0.0489 (9)	0.0534 (9)	0.0048 (7)	0.0012 (7)	0.0274 (7)
C28	0.0450 (9)	0.0517 (9)	0.0540 (9)	0.0097 (7)	0.0087 (7)	0.0256 (8)
C29	0.0385 (8)	0.0576 (10)	0.0636 (11)	0.0048 (7)	0.0055 (7)	0.0270 (8)
C30	0.0586 (12)	0.0989 (16)	0.0749 (13)	0.0302 (11)	0.0247 (10)	0.0333 (12)
N3	0.0459 (8)	0.0723 (10)	0.0775 (11)	0.0128 (7)	0.0157 (8)	0.0415 (9)
O9	0.0515 (7)	0.0899 (9)	0.0549 (7)	0.0189 (6)	0.0122 (6)	0.0260 (7)
O10	0.0462 (7)	0.0841 (9)	0.0559 (7)	0.0035 (6)	−0.0022 (6)	0.0309 (7)
O11	0.0399 (7)	0.1124 (12)	0.0902 (10)	0.0106 (7)	0.0049 (7)	0.0505 (9)
O12	0.0636 (9)	0.1155 (13)	0.0860 (11)	0.0232 (8)	0.0337 (8)	0.0380 (9)

### Geometric parameters (Å, º)

**Table:**

C1—C2	1.285 (4)	C16—N2	1.448 (2)
C1—H1A	0.9300	C17—O6	1.345 (2)
C1—H1B	0.9300	C17—C18	1.411 (2)
C2—C3	1.460 (4)	C18—O5	1.359 (2)
C2—H2	0.9300	C18—C19	1.374 (2)
C3—C4	1.512 (3)	C19—H19	0.9300
C3—H3A	0.9700	C20—O5	1.423 (2)
C3—H3B	0.9700	C20—H20A	0.9600
C4—C5	1.363 (3)	C20—H20B	0.9600
C4—C9	1.404 (3)	C20—H20C	0.9600
C5—C6	1.393 (3)	N2—O8	1.219 (2)
C5—H5	0.9300	N2—O7	1.240 (2)
C6—C7	1.392 (3)	O6—H6	0.83 (2)
C6—N1	1.449 (2)	C21A—C22A	1.206 (8)
C7—O2	1.344 (2)	C21A—H21A	0.9300
C7—C8	1.412 (2)	C21A—H21B	0.9300
C8—O1	1.355 (2)	C22A—C23	1.475 (5)
C8—C9	1.370 (2)	C22A—H22A	0.9300
C9—H9	0.9300	C21B—C22B	1.32 (2)
C10—O1	1.432 (2)	C21B—H21C	0.9300
C10—H10A	0.9600	C21B—H21D	0.9300
C10—H10B	0.9600	C22B—C23	1.320 (9)
C10—H10C	0.9600	C22B—H22B	0.9300
N1—O4	1.224 (2)	C23—C24	1.520 (3)
N1—O3	1.247 (2)	C23—H23A	0.9700
O2—H2O	0.91 (3)	C23—H23B	0.9700
C11A—C12A	1.293 (12)	C23—H23C	0.9700
C11A—H11A	0.9300	C23—H23D	0.9700
C11A—H11B	0.9300	C24—C25	1.373 (2)
C12A—C13	1.383 (5)	C24—C29	1.402 (3)
C12A—H12A	0.9300	C25—C26	1.395 (2)
C11B—C12B	1.39 (2)	C25—H25	0.9300
C11B—H11C	0.9300	C26—C27	1.389 (2)
C11B—H11D	0.9300	C26—N3	1.450 (2)
C12B—C13	1.351 (9)	C27—O10	1.341 (2)
C12B—H12B	0.9300	C27—C28	1.415 (2)
C13—C14	1.520 (3)	C28—O9	1.359 (2)
C13—H13A	0.9700	C28—C29	1.370 (2)
C13—H13B	0.9700	C29—H29	0.9300
C13—H13C	0.9700	C30—O9	1.423 (2)
C13—H13D	0.9700	C30—H30A	0.9600
C14—C15	1.364 (3)	C30—H30B	0.9600
C14—C19	1.404 (3)	C30—H30C	0.9600
C15—C16	1.397 (3)	N3—O12	1.219 (2)
C15—H15	0.9300	N3—O11	1.240 (2)
C16—C17	1.392 (2)	O10—H10	0.86 (3)
			
C2—C1—H1A	120.0	O6—C17—C16	126.40 (16)
C2—C1—H1B	120.0	O6—C17—C18	116.84 (15)
H1A—C1—H1B	120.0	C16—C17—C18	116.75 (15)
C1—C2—C3	127.5 (3)	O5—C18—C19	125.52 (16)
C1—C2—H2	116.2	O5—C18—C17	114.12 (14)
C3—C2—H2	116.2	C19—C18—C17	120.36 (16)
C2—C3—C4	113.5 (2)	C18—C19—C14	121.83 (17)
C2—C3—H3A	108.9	C18—C19—H19	119.1
C4—C3—H3A	108.9	C14—C19—H19	119.1
C2—C3—H3B	108.9	O5—C20—H20A	109.5
C4—C3—H3B	108.9	O5—C20—H20B	109.5
H3A—C3—H3B	107.7	H20A—C20—H20B	109.5
C5—C4—C9	118.99 (18)	O5—C20—H20C	109.5
C5—C4—C3	121.19 (18)	H20A—C20—H20C	109.5
C9—C4—C3	119.81 (18)	H20B—C20—H20C	109.5
C4—C5—C6	119.87 (17)	O8—N2—O7	121.72 (18)
C4—C5—H5	120.1	O8—N2—C16	119.09 (19)
C6—C5—H5	120.1	O7—N2—C16	119.18 (17)
C7—C6—C5	122.23 (16)	C18—O5—C20	117.02 (14)
C7—C6—N1	120.24 (18)	C17—O6—H6	101.5 (17)
C5—C6—N1	117.52 (17)	C22A—C21A—H21A	120.0
O2—C7—C6	125.82 (16)	C22A—C21A—H21B	120.0
O2—C7—C8	116.97 (16)	H21A—C21A—H21B	120.0
C6—C7—C8	117.20 (16)	C21A—C22A—C23	132.2 (6)
O1—C8—C9	125.18 (16)	C21A—C22A—H22A	113.9
O1—C8—C7	114.62 (16)	C23—C22A—H22A	113.9
C9—C8—C7	120.19 (16)	C22B—C21B—H21C	120.0
C8—C9—C4	121.50 (17)	C22B—C21B—H21D	120.0
C8—C9—H9	119.3	H21C—C21B—H21D	120.0
C4—C9—H9	119.3	C23—C22B—C21B	143.2 (12)
O1—C10—H10A	109.5	C23—C22B—H22B	108.4
O1—C10—H10B	109.5	C21B—C22B—H22B	108.4
H10A—C10—H10B	109.5	C22B—C23—C24	120.4 (4)
O1—C10—H10C	109.5	C22A—C23—C24	115.5 (2)
H10A—C10—H10C	109.5	C22A—C23—H23A	108.4
H10B—C10—H10C	109.5	C24—C23—H23A	108.4
O4—N1—O3	121.93 (19)	C22A—C23—H23B	108.4
O4—N1—C6	119.1 (2)	C24—C23—H23B	108.4
O3—N1—C6	118.96 (19)	H23A—C23—H23B	107.5
C8—O1—C10	117.66 (15)	C22B—C23—H23C	107.2
C7—O2—H2O	105.1 (17)	C24—C23—H23C	107.2
C12A—C11A—H11A	120.0	C22B—C23—H23D	107.2
C12A—C11A—H11B	120.0	C24—C23—H23D	107.2
H11A—C11A—H11B	120.0	H23C—C23—H23D	106.9
C11A—C12A—C13	126.5 (7)	C25—C24—C29	118.75 (16)
C11A—C12A—H12A	116.8	C25—C24—C23	120.96 (18)
C13—C12A—H12A	116.8	C29—C24—C23	120.28 (17)
C12B—C11B—H11C	120.0	C24—C25—C26	119.89 (16)
C12B—C11B—H11D	120.0	C24—C25—H25	120.1
H11C—C11B—H11D	120.0	C26—C25—H25	120.1
C13—C12B—C11B	134.0 (11)	C27—C26—C25	122.34 (15)
C13—C12B—H12B	113.0	C27—C26—N3	120.08 (15)
C11B—C12B—H12B	113.0	C25—C26—N3	117.58 (15)
C12B—C13—C14	118.1 (3)	O10—C27—C26	126.64 (15)
C12A—C13—C14	116.4 (2)	O10—C27—C28	116.39 (15)
C12A—C13—H13A	108.2	C26—C27—C28	116.96 (14)
C14—C13—H13A	108.2	O9—C28—C29	125.80 (15)
C12A—C13—H13B	108.2	O9—C28—C27	113.62 (14)
C14—C13—H13B	108.2	C29—C28—C27	120.58 (16)
H13A—C13—H13B	107.3	C28—C29—C24	121.47 (15)
C12B—C13—H13C	107.8	C28—C29—H29	119.3
C14—C13—H13C	107.8	C24—C29—H29	119.3
C12B—C13—H13D	107.8	O9—C30—H30A	109.5
C14—C13—H13D	107.8	O9—C30—H30B	109.5
H13C—C13—H13D	107.1	H30A—C30—H30B	109.5
C15—C14—C19	118.49 (17)	O9—C30—H30C	109.5
C15—C14—C13	121.91 (18)	H30A—C30—H30C	109.5
C19—C14—C13	119.59 (18)	H30B—C30—H30C	109.5
C14—C15—C16	120.02 (17)	O12—N3—O11	121.95 (16)
C14—C15—H15	120.0	O12—N3—C26	119.16 (16)
C16—C15—H15	120.0	O11—N3—C26	118.88 (16)
C17—C16—C15	122.55 (16)	C28—O9—C30	117.87 (14)
C17—C16—N2	119.58 (16)	C27—O10—H10	102.6 (17)
C15—C16—N2	117.87 (16)		
			
C1—C2—C3—C4	−120.5 (3)	O6—C17—C18—C19	179.56 (15)
C2—C3—C4—C5	95.0 (3)	C16—C17—C18—C19	0.2 (2)
C2—C3—C4—C9	−84.5 (3)	O5—C18—C19—C14	179.73 (17)
C9—C4—C5—C6	0.1 (3)	C17—C18—C19—C14	0.1 (3)
C3—C4—C5—C6	−179.48 (18)	C15—C14—C19—C18	−0.1 (3)
C4—C5—C6—C7	−0.3 (3)	C13—C14—C19—C18	179.3 (2)
C4—C5—C6—N1	179.90 (16)	C17—C16—N2—O8	−173.68 (18)
C5—C6—C7—O2	−178.92 (16)	C15—C16—N2—O8	6.3 (3)
N1—C6—C7—O2	0.8 (3)	C17—C16—N2—O7	6.4 (3)
C5—C6—C7—C8	−0.3 (2)	C15—C16—N2—O7	−173.57 (17)
N1—C6—C7—C8	179.51 (14)	C19—C18—O5—C20	1.2 (3)
O2—C7—C8—O1	−0.1 (2)	C17—C18—O5—C20	−179.13 (16)
C6—C7—C8—O1	−178.89 (14)	C21B—C22B—C23—C24	−92.5 (19)
O2—C7—C8—C9	179.89 (15)	C21A—C22A—C23—C24	113.8 (7)
C6—C7—C8—C9	1.1 (2)	C22B—C23—C24—C25	2.5 (7)
O1—C8—C9—C4	178.59 (16)	C22A—C23—C24—C25	−45.2 (4)
C7—C8—C9—C4	−1.4 (3)	C22B—C23—C24—C29	−176.1 (7)
C5—C4—C9—C8	0.8 (3)	C22A—C23—C24—C29	136.1 (3)
C3—C4—C9—C8	−179.65 (18)	C29—C24—C25—C26	−0.2 (3)
C7—C6—N1—O4	−176.42 (16)	C23—C24—C25—C26	−178.83 (18)
C5—C6—N1—O4	3.4 (2)	C24—C25—C26—C27	−0.1 (3)
C7—C6—N1—O3	4.4 (3)	C24—C25—C26—N3	179.01 (15)
C5—C6—N1—O3	−175.79 (17)	C25—C26—C27—O10	−178.86 (15)
C9—C8—O1—C10	−2.6 (3)	N3—C26—C27—O10	2.0 (2)
C7—C8—O1—C10	177.44 (16)	C25—C26—C27—C28	0.4 (2)
C11B—C12B—C13—C14	100.3 (13)	N3—C26—C27—C28	−178.66 (14)
C11A—C12A—C13—C14	−105.3 (7)	O10—C27—C28—O9	−1.6 (2)
C12B—C13—C14—C15	175.2 (5)	C26—C27—C28—O9	179.02 (14)
C12A—C13—C14—C15	−132.1 (3)	O10—C27—C28—C29	178.92 (15)
C12B—C13—C14—C19	−4.2 (5)	C26—C27—C28—C29	−0.5 (2)
C12A—C13—C14—C19	48.6 (4)	O9—C28—C29—C24	−179.25 (16)
C19—C14—C15—C16	−0.1 (3)	C27—C28—C29—C24	0.1 (3)
C13—C14—C15—C16	−179.5 (2)	C25—C24—C29—C28	0.2 (3)
C14—C15—C16—C17	0.3 (3)	C23—C24—C29—C28	178.83 (19)
C14—C15—C16—N2	−179.68 (17)	C27—C26—N3—O12	−179.78 (16)
C15—C16—C17—O6	−179.69 (16)	C25—C26—N3—O12	1.1 (2)
N2—C16—C17—O6	0.3 (3)	C27—C26—N3—O11	1.1 (2)
C15—C16—C17—C18	−0.4 (2)	C25—C26—N3—O11	−178.04 (16)
N2—C16—C17—C18	179.66 (15)	C29—C28—O9—C30	−3.2 (3)
O6—C17—C18—O5	−0.1 (2)	C27—C28—O9—C30	177.35 (16)
C16—C17—C18—O5	−179.54 (14)		

### Hydrogen-bond geometry (Å, º)

**Table:**

*D*—H···*A*	*D*—H	H···*A*	*D*···*A*	*D*—H···*A*
O10—H10···O11	0.86 (3)	1.81 (3)	2.594 (2)	149 (2)
O6—H6···O7	0.83 (2)	1.83 (2)	2.584 (2)	152 (2)
O2—H2*O*···O3	0.91 (3)	1.78 (3)	2.587 (2)	146 (2)
C12*A*—H12*A*···O12	0.93	2.58	3.382 (4)	145
C12*B*—H12*B*···O3^i^	0.93	2.56	3.325 (8)	140
C9—H9···O7^ii^	0.93	2.59	3.394 (2)	145

Symmetry codes: (i) −*x*+1, −*y*+1, −*z*+1; (ii) −*x*+2, −*y*+1, −*z*+2.
